# Supplementary material for: Ischemic Stroke Risk Associated with Mitochondrial Haplogroup F in the Asian Population
Source: Cells. 2020 Aug 11;9(8):1885. doi: 10.3390/cells9081885 (PMC7463505; doi:10.3390/cells9081885)
Supplement: Supplementary file 1 [file cells-09-01885-s001.pdf]

Supplemental tables and figures

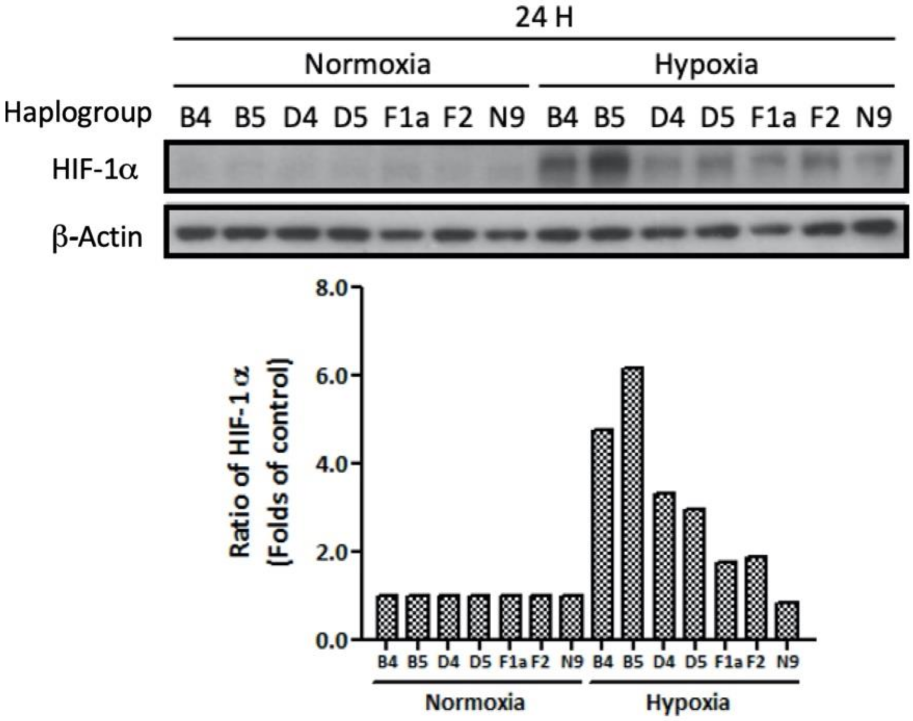

Supplemental Figure S1: HIF-1α expressions in different cybrids under normoxic and hypoxic conditions. Twenty-four hours after hypoxic treatment, discrepant expressions of HIF-1α was observed in cybrid harboring different mtDNA haplogroup (B4, B5, D4, D5, F1a, F2, N9). β-actin was loading control.

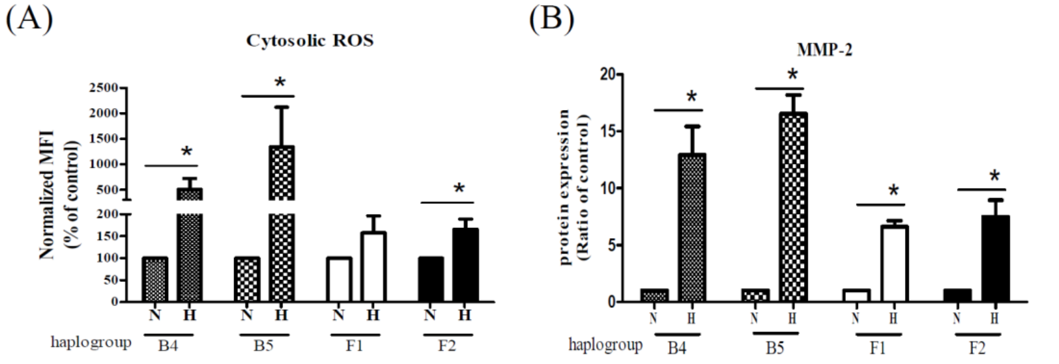

Supplemental Figure S2: Comparisons of intracellular ROS generation (**A**), induction of MMP-2 (**B**) in different cybrids under hypoxia-ischemia. DCF-based flow cytometry was used for detecting cytosolic ROS. Protein expression of MMP-2 was assessed by western blot. All data are shown as means ± S.D. (n = 4). *$p < 0.05$. N was used to represent normoxic and H to represent hypoxic condition for 24 hours.

Supplemental Table S1: 24 pair primers for the mitochondrial DNA to perform the gene amplification by polymerase chain reaction.

	**primer**	**sequence (5'→3')'**		**sequence (3'→5')'**	**PCR product**
**1**	631	ACATCACCCCATAAACAAATAggTT	931	gCTTCTATTgACTTgggTTAATCg	301
**2**	1272	AgCAAACCCTgATgAAggCTAC	1781	TATATCTATTgCgCCAggTTTCAAT	510
**3**	2698	AgAggCgggCATgACACAgCA	3066	gATCACgTAggACTTTAATCgTTgA	369
**5**	3611	TCCTATTTATTCTAgCCACCTCTAg	3862	ATCATATTATggCCAAgggTCATg	252
**6**	3916	gAgTCCgAACTAgTCTCAggCT	4255	gAgggggAATgCTggAgATTgTA	340
**7**	4344	TCgAACCCATCCCTgAgAATCC	4577	gTTTATTTCTAggCCTACTCAggTAA	234
**8**	4623	TCCACAgAAgCTgCCATCAAgTA	4940	gAgAgTgAggAgAAggCTTACgT	318
**9**	4989	CAgCTACgCAAAATCTTAgCATAC	5257	TTgggCAAAAAgCCggTTAgCg	269
**10**	5921	ACTATTCTCTACAAACCACAAAgAC	6284	TgTTCAACCTgTTCCTgCTCCg	364
**11**	6535	CAgACCgCAACCTCAACACCAC	6807	gTgTgTCTACgTCTATTCCTACTg	273
**12**	8153	ggggTATACTACggTCAATgCTC	8530	TCATTTTggTTCTCAgggTTTgTTAT	378
**13**	8628	CAAATATCTCATCAACAACCgACTA	8994	CAgggCTATTggTTgAATgAgTAg	367
**14**	9044	TAATTggAAgCgCCACCCTAgC	9414	ggCCTTggTATgTgCTTTCTCgT	371
**15**	9673	gAAACCAAATAATTCAAgCACTgCT	9987	ACCCTCATCAATAgATggAgACAT	315
**16**	10277	ACCCCTACCATgAgCCCTACAA	10515	gTgAgATggTAAATgCTAgTATAATAT	239
**17**	12274	AggATAACAgCTATCCATTggTCT	12545	gTggCTCAgTgTCAgTTCgAgAT	272
**18**	12582	AgACTACTTCTCCATAATATTCATCC	12858	gTATAggATTgCTTgAATggCTgC	277
**19**	13077	CCACTCAAgCACTATAgTTgTAgC	13591	TCAgggAggTAgCgATgAgAgTA	515
**20**	13711	gCCggAAgCCTATTCgCAggAT	13980	CAggTTTTggCTCgTAAgAAggC	270
**21**	14217	CTAATCAACgCCCATAATCATACAA	14562	gTCgggTgTgTTATTATTCTgAATTT	346
**22**	14829	TCCgCATgATgAAACTTCggCT	15175	ggCCCCTCAgAATgATATTTggC	347
**23**	15257	gACAgTCCCACCCTCACACgAT	15600	gggACggATCggAgAATTgTgT	344
**24**	15696	TTCgCCCACTAAgCCAATCACTT	16037	TCCCCATgAAAgAACAgAgAATAgT	342

Supplemental Table S2: The oligonucleotide probes sequences for mtDNA haplogrouping

Probe for hybridization				Probe for hybridization		
position	purpose	sequence (5'→3')		position	purpose	sequence (5'→3')
663	a	GCTAATAGAAAGGCCAGGA		8701	a	AGTGTTGTGTATGGCTATCATT
681	a	TGTAATCTTACTGAGAGCTAAT		8701	b(N)	AGTGTTGTGTATGGTTATCAT
681	b	TGTAATCTTACTAAGAGCTAA		8793	a	CGGACTCCTGCCCCACTCA
752	a	CGTGCTTGATGCTTATTCCTTTTGA		8794	a	TGGTGTAAATGAGTAAGGCAGG
1382	a	GGCTATCGTAGTTGTCTGGG		9123	a	GATTTCTAGGATAGTTAGTAGAAT
1438	a	AGCACTCTACTCTTAGTTTACT		9219	p	ATCACATGCCTATCATATAGTA
1442	a	AACTAAGCACTCTATTCTCAGT		9296	a	CTAATGACCTCCGGTCTAGCC
1647	p	AGGAGATTTCAACTTAACTTGA		9452	a	TTCGATACGGAATAATCCTATT
3010	a	ATCAGGACATCCCAATGGTG		9774	p	ATGCCGTCGGAAATGGTGA
3010	b	ATCAGGACATCCCGATGGT		9950	a	ATTTTGTAGATGTGGTCTGACTA
3027	a	TGCAGCCGCTATCAAAGG		9968	a	GACTATTTCTGTATGTTTC
3644	a	GGATTGAGTAAGCGGCT		10310	a	ACTATTAGTGGTAGGTTAGTT
3667	p	TAGTTTGAGTTTGATGCTCA		10310	b	TATTAGTGGCAGGTTAGTTGTT
3714	a	GAGATTGTTTGGGCCACTGCT		10320	a	CCACTAATAATTATGTCATCCC
3714	b	GAGATTGTTTGGGCTACTGCT		10397	a	TACCAATTCAGCCCAGTCTAAT
3970	a	GGCTATGAAGAATAAGGCGA		10400	m	TACCAATTCAGCTCAGTCT
4048	a	CTAGGAACAACATATAACGCACTC		10400	m-1	ACTATATACCAATTCAGCTCAGT
4048	b	CTAGGAACAACATATGACGCACTC		10400	n-1	ACTATATACCAATTCGGTTCAGT
4071	a	GACAAAATATGTTGTATAGAGTTC		10454	a	GACTCATTAAATTATGACAATCATA
4071	b	ACAAAATATGTTGTGTAGAGTTC		12338	a	AAGTAACAACCATGCACACTA
4086	a	AAGTAGGGTCTTGGTAACAAAATA		12372	a	TAACCCTAACTTCCCTAATTC
4386	a	GGTGTGGTAGGTGGCAC		12406	a	ATCCTTACCACCCTCATTAACC
4386	b	GGGTGTGATAGGTGGC		12501	a	CAACAATATTCATATGCCTAG
4491	a	CTGGCCCAACCCATCATCTA		12501	b	ACAACAATATTCATGTGCCT
4715	a	TTATGGTTCATTGCCCGGAG		12630	a	CGTTACATGATCCATCATAGA
4820	a	TTCTGAGTCCCAGAAGTTACCC		12630	a	CGTTACATGATCCATCATAGA
4833	a	AGGTTACCCAAGGCGCCCCT		12705	a	CTACTCATCTTCCTAATTACC
4850	a	CTGACATCCGGTCTGCTT		12775	p	TAGGAATTATATCCTTCTTGC
4883	a	AACTAGCCCCTATCTCAAT		13156	p	CTAAGCATAGTGTTAGAGTTTG
4895	a	CCATCTCAATCATGTACCAA		13263	a	TATTATGAGTCCTAGCTGACTTG
4895	b	ATCTCAATCATATACCAAATC		13928	a	GGATTCTACCCTACCATCA
5108	a	TTATCCTAACTACCACCGCA		13928	b	GATTCTACCCTAGCATCA
5147	a	CTCCAGCACCACAACC		13942	a	ATCACACACCGCGCAAT
5178	a	TGAAACAAGATAACATGAC		14308	a	TAATAGTGTAGGGAGCTGAAT
5178	b	CTGAAACAAGCTAACATGA		14343	p	GTGGGTGAAAGAGTATGATG
5231	a	TCCCTAGGAGGCCTACCCCC		14476	a	CTGTAGTATATCCAAAAACAACC
5301	a	TAGCCTCATCGTCCCCACCAT		14569	a	ACACACCCGACCACACCACTAA
6146	a	GCTTTGGCAACTGGCTAGTTC		14893	a	TGCATGGCTAGGAACAGTCCT
6179	a	GTGCCCCCGATATAGCGTTT		14893	b	GCATGGCTAGGAATAGTCC
6680	a	CTCCCATATTGTAACCTACTACT		14979	a	AAGGTAGCGGATGGTTCAGC
6752	a	CAATTGGCTTCCTGGGGTT		15346	a	CACCTCCTATTCTTACACGAAA
6752	b	CAATTGGCTTCCTAGGGTTT		15440	a	ACGCCCTCGGCCTACTTCT
8272	a	TGGGCTCTAGAGGTGGTGCTAT		15487	a	TATTCTCACCTGACCTCCT
8272	b	CTCTAGAGGGGGTAGAGGTGGTGCT		15524	a	TTATACCCTAGCCGACC
8392	a	GTAATTATGGTGGGTCATACG		15535	a	CCAACCCCTTAAATACCCCTC
8584	a	TACCCGCCACAGTACTGATCA		15535	b	AACCCCTTAAACACCCCTCC
8684	a	ATCATTTGTTTTGAGATTAGTTT		15924	a	GTTTTCATCTCCGGCTTACAAG

Supplemental Table S3: Summary of the major non-synonymous mtDNA variants in the coding region of various mitochondrial haplogroups.

mtDNA SNP (A-A change)	Locus	Macro-haplogroup N						Macro-haplogroup M							
		**A**	**B4**	**B5**	**F1**	**F2**	**N9a**	**C**	**D4**	D5	E	G	M7b	M7c	M8a
		(*n* = 5)	(*n* = 10)	(*n* = 10)	(*n* = 10)	(*n* = 10)	(*n* = 5)	(*n* = 5)	(*n* = 10)	(*n* = 10)	(*n* = 5)	(*n* = 5)	(*n* = 10)	(*n* = 10)	(*n* = 5)
**G4048A (Asp-Asn)**	ND1	—	—	—	—	—	—	—	—	—	—	—	—	80% A	—
A4824G (Thr-Ala)	ND2	100% G	—	—	—	—	—	—	—	—	—	—	—	—	—
A4833G (Thr-Ala)	ND2	—	—	—	—	—	—	—	—	—	—	100% G	—	—	—
C5178A (Leu-Met)	ND2	—	—	—	—	—	—	—	100% A	100% A	—	—	—	—	—
A5301G (Ile-Val)	ND2	—	—	—	—	—	—	—	—	100% G	—	—	—	—	—
T5442C (Phe-Leu)	ND2	—	—	—	—	—	—	—	—	—	—	—	—	100% C	—
G5460A (Ala-Thr)	ND2	—	—	—	—	—	—	—	—	—	—	—	—	80% A	—
G7598A (Ala-Thr)	COX2	—	—	—	—	—	—	—	—	—	100% A	—	—	—	—
G7853A (Val-Ile)	COX2	—	—	—	—	—	—	—	—	—	—	—	90% A	—	—
G8584A (Ala-Thr)	ATP6	—	—	100% A	—	—	—	80% A	—	—	—	—	—	—	80% A
C8684T (Thr-Ile)	ATP6	—	—	—	—	—	—	—	—	—	—	—	—	—	100% T
A8701G (Thr-Ala)	ATP6	—	—	—	—	—	—	100% G	100% G	100% G	100% G	100% G	100% G	100% G	100% G
C8794T (His-Tyr)	ATP6	100% T	—	—	—	—	—	—	—	—	—	—	—	—	—
G9053A (Ser-Asn)	ATP6	—	—	—	100% G	—	—	—	—	—	—	—	—	—	—
A10398G (Thr-Ala)	ND3	—	—	100% G	—	—	—	100% G	100% G	100% G	100% G	100% G	100% G	100% G	100% G
T10609C (Met-Thr)	ND4L	—	—	—	100% G	—	—	—	—	—	—	—	—	—	—
G11969A (Ala-Thr)	ND4	—	—	—	—	—	—	100% A	—	—	—	—	—	—	—
T12338C (Met-Thr)	ND5	—	—	—	—	100% C	—	—	—	—	—	—	—	—	—
A12358G (Thr-Ala)	ND5	—	—	—	—	—	100% G	—	—	—	—	—	—	—	—
G12406A (Val-Ile)	ND5	—	—	—	100% A	—	—	—	—	—	—	—	—	—	—
T12811C (Tyr-His)	ND5	—	—	—	—	—	—	—	—	—	—	—	80% C	—	—
A13263G (Gln-His)	ND5	—	—	—	—	—	—	100% G	—	—	—	—	—	—	—
G13708A (Ala-Thr)	ND5	—	—	—	—	100% A	—	—	—	—	—	—	—	—	—
G13759A (Ala-Thr)	ND5	—	—	—	100% A	—	—	—	—	—	—	—	—	—	—
G13928C (Ser-Asn)	ND5	—	—	—	100% C	100% C	—	—	—	—	—	—	—	—	—
T14318C (Asn-Ser)	ND5	—	—	—	—	—	—	100% C	—	—	—	—	—	—	—
T15204C (Ile-Thr)	CytB	—	—	—	—	—	—	100% C	—	—	—	—	—	—	—

SNP: single nucleotide polymorphism; A-A: amino acid; "—" denotes 100% wild-type; Ala: Alanine; Asn: asparagine; Asp: aspartic acid; Gln: glutamine; His: histidine;. Ile: isoleucine; Leu: leucine; Met: methionine; Phe: phenylalanine; Ser: serine; Thr: threonine; Tyr: tyrosine; Val: valine; ND1~5: NADH dehydrogenase subunit 1~5; ATP6: ATP synthase 6; COX2: cytochrome c oxidase subunit II; CytB: cytochrome b.
